# Epidemiological Determinants of COVID-19-Related Patient Outcomes in Different Countries and Plan of Action: A Retrospective Analysis

**DOI:** 10.7759/cureus.8440

**Published:** 2020-06-04

**Authors:** Sayak Roy, Maneesha Khalse

**Affiliations:** 1 Internal Medicine, Medica Superspeciality Hospital, Kolkata, IND; 2 Medical Services, Medical Affairs Division, Lupin Limited, Mumbai, IND

**Keywords:** covid-19, covid-19 prevention, epidemiological determinants

## Abstract

Current development around the pandemic of novel coronavirus disease 2019 (COVID-19) presents a significant healthcare resource burden threatening to overwhelm the available nationwide healthcare infrastructure. It is essential to consider, especially for resource-limited nations, strategizing the coordinated response to handle this crisis effectively and preparing for the upcoming emergence of calamity caused by this yet-to-know disease entity.

Relevant epidemiological data were retrieved from currently available online reports related to COVID-19 patients. The correlation coefficient was calculated by plotting dependant variables - the number of COVID-19 cases and the number of deaths due to COVID 19 on the Y-axis and independent variables - critical-care beds per capita, the median age of the population of the country, the number of COVID-19 tests per million population, population density (persons per square km), urban population percentage, and gross domestic product (GDP) expense on health care - on the X-axis.

After analyzing the data, both the fatality rate and the total number of COVID-19 cases were found to have an inverse association with the population density with the variable - the number of cases of COVID-19 - achieving a statistical significance (p-value 0.01). The negative correlation between critical care beds and the fatality rate is well-justified, as intensive care unit (ICU) beds and ventilators are the critical elements in the management of complicated cases. There was also a significant positive correlation between GDP expenses on healthcare by a country and the number of COVID-19 cases being registered (p-value 0.008), although that did not affect mortality (p-value 0.851).

This analysis discusses the overview of various epidemiological determinants possibly contributing to the variation in patient outcomes across regions and helps improve our understanding to develop a plan of action and effective control measures in the future.

## Introduction

A significant epidemic focus of new coronavirus disease 2019 (COVID-19) was identified in December 2019 in Wuhan, China, which rapidly progressed across 176 countries in Europe, North America, Asia, and the Middle East, affecting more than half a million people [1]. Later, this disease outbreak, caused by a novel coronavirus SARS-CoV-2 with an unknown origin, was declared as a significant pandemic by the World Health Organisation (WHO) on March 11, 2020. Europe has become the new major epicenter with the total number of cases and deaths being reported as 686,338 and 52,809, respectively [2]. This is the third coronavirus outbreak with a novel strain in the last two decades and presents an ensuing healthcare resource burden that threatens to overwhelm available healthcare resources [3]. As a result, the challenges presented seem to be unique in the disease prospect, considering disparate resource settings across countries, especially when applying strategies from high-technology intensive care settings to less developed areas. The global burden of COVID-19, which is an infectious agent with high transmissibility and a moderate fatality rate, is likely to fall hardest on the vulnerable groups in low middle-income countries (LMICs). Therefore, a systematic strengthening in its ability from the technical and financial fronts is warranted to respond to this challenging situation successfully. Public health measures, such as surveillance, exhaustive contact tracing, social distancing, travel restrictions, public education on hand hygiene, ensuring flu vaccinations for the frail and immunocompromised, and temporarily suspending non-essential surgical procedures and services will play their part in delaying the spread of infection and dispersing pressure on hospitals [4]. SARS-CoV-2 is likely to play havoc on the world economy, heading to an apprehended shrinkage of the global economy in 2020 by 1% [5]. In this brief analysis, we tried to overview various socioeconomic determinants possibly contributing to variation in COVID-19-related outcomes across regions and then make a plan of action based on the evidence.

Given the unpredictable course of this global crisis, infection and mortality rates vary widely from one country to another. Apart from the baseline demographic features of patients, socioeconomic factors, such as income groups, population density, access to health care, and quality of health system resources, may account for the observed variations in mortality rates. Different testing strategies, reporting systems, and data availability play an essential part in these highly variable statistics, even with the number of unreported cases believed to be quite considerable in some countries.

The total number of cases worldwide is 750,890, and the disease has caused 36,405 (4.84%) deaths from December 29, 2019, to March 31, 2020 [6]. However, the highest mortality was found in Italy, Spain, United Kingdom, France, and Iran. The overall case fatality rate of March 23 in Italy (7.2%) is substantially higher than in China (2.3%). Now, the fatality rate of Italy has increased to 13.3% as of April 21 (number of cases recorded 181,228 and number of deaths 24,114), which is closely followed by the United Kingdom with a fatality rate of 13.23% (number of COVID-19 cases being 124,743 and number of deaths being 16,509) [6-7]. This feature demonstrates the daily change in the fatality rate in a pandemic. Moreover, the infections occurred predominantly (87%) in people 30 to 79 years old. The most likely populations requiring mechanical ventilation are the elderly and people with pre-existing comorbidities (in particular, cardiovascular disease and hypertension, followed by diabetes mellitus) with a predicted mortality of around 15% to 49% [8].

Despite the known impact of per capita expenditure on health care and reduced mortality in previous influenza pandemics (2009), the findings in the recent outbreak entail no significant correlation between health care spending and the COVID-19-related mortality observed in the population [9]. In particular, the mortality rates are highly variable in different high-income countries, as noticed in Europe and the USA based on their health spending.

As per the World Bank statistics, the US spent 17.8% of its gross domestic product (GDP) on health care while other countries, including India, are lagging behind primarily due to the income category of these countries [10]. Surprisingly, this did not affect the total number of cases infected with COVID-19 [2].

This consistent observation was reported in a descriptive analysis study (in preprint). In that study, there was no significant correlation between the GDP growth of the country and the number of treating physicians/1000 patient population with any COVID-19-related outcome but a negative correlation between COVID-19-related deaths and the number of beds available per 1000 population. Additionally, there was an inverse correlation between the number of tests conducted per million population with the rates of active infections, new cases, and new deaths due to COVID-19 [11]. Herein, we try to analyze the impact of various socioeconomic and demographic features of a few selected countries, namely, United States of America (USA), Germany, Italy, France, South Korea, Spain, Japan, United Kingdom (UK), China, and India, concerning COVID-19-related cases and fatality rates.

## Materials and methods

We retrieved data between January 2020 and April 8, 2020, related to population and population density, the median age of the population of a country, urban population, number of COVID-19 testing employed per million population, GDP expense of each country on health, critical care beds available per capita, from various sources as mentioned in the reference section, stated next to each of these variables in Table 1, along with the total number of COVID-19 cases and the case fatality rate (as per WHO Situation Report, 78 [2]).

**Table 1:** Baseline regional characteristics of population 2019, health care expense, critical-care beds per capita, total cases, total number of tests conducted, and case fatality rate of COVID-19 GDP: gross domestic product

Country name	Population 2019 [12] (millions)	Population density (persons per square km) [13]	Urban population (%) [13]	Median age of population [13] (years)	Critical-care beds Per Capita(per 100,000 inhabitants of the country) [14]	GDP expense on Health care [15](%)	Total cases due to COVID-19 [16]	Number of COVID-19 tests per million population [17]	Case fatality rate COVID-19 [17](%)
USA	331	36	83	38	34.7	17.1	333811	6,336	2.86
Germany	80.3	230.3	76	46	29.2	11.1	99225	10,962	1.62
Italy	62.3	211.9	69	47	12.5	8.9	132547	12,495	12.46
France	67.6	105.6	82	42	11.6	11.5	73488	3,436	12.10
South Korea	51.6	532.8	82	44	10.6	5	10331	9,310	1.85
Spain	49.7	99.6	80	45	9.7	9	135032	7,593	9.66
Japan	125.9	345.3	92	48	7.3	10.9	3906	437	2.04
United Kingdom	65.4	270.5	83	40	6.6	9.8	51612	3,929	10.41
China	1400	149	61	38	3.6	9	83071	2831	4.02
India	1300	441.1	35	28	2.3	3.7	4067	102	2.68

We then applied Pearson’s correlation coefficient to see the correlation of various demographic features and COVID-19 cases and deaths due to COVID-19 using the online calculator available, https://www.socscistatistics.com/tests/pearson/default2.aspx [18].

## Results

The correlations between population characteristics and socioeconomic variables in various countries as discussed earlier with respect to outcome in terms of total positive cases and fatality rate due to COVID-19 are summarized in Table 2.

**Table 2:** Correlation of various baseline independent variables on the X-axis v/s dependent variables - case fatality rate and total COVID-19 cases on the Y-axis (Pearson’s correlation coefficient) *statistically significant (p<0.05) GDP: gross domestic product

Baseline socioeconomic variables (X-axis) v/s case fatality rate of COVID-19 cases (Y-axis)		
	R-value	P-value
Critical-care beds per capita	-0.237	0.509
Median age of the population of the country	0.2104	0.56
Number of COVID-19 tests per million population	0.193	0.59
Population density (persons per square km)	-0.43	0.21
Urban population percentage	0.137	0.705
GDP expense on health care	0.068	0.851
Baseline socioeconomic variables (X-axis) v/s total COVID-19 positive cases (Y-axis)		
Critical-care beds per capita	0.76	0.009*
Median age of population of the country	0.21	0.56
Number of COVID-19 tests per million population	0.356	0.31
Population density (persons per square km)	-0.753	0.01*
Urban population percentage	0.212	0.556
GDP expense on health care	0.777	0.008*

There is a strong positive correlation between GDP expense on the health of a country with the number of cases getting detected. The reason for this is the affordability of easily testing a higher number of patients in high-income countries; however, this expense, on the other hand, did not show any significance with deaths due to COVID-19 (p-value 0.851).

## Discussion

The result showed that both the case fatality rate and the number of COVID-19 cases are negatively correlated to population density, which seems quite strange. However, on further analysis, by taking the USA and the European countries only on the X-axis and case fatality from these countries on the Y-axis, the same Pearson’s correlation coefficient r-value becomes 0.168 (p-value 0.75), which is now positively related and not statistically significant after adjustment. The same calculation using the population density of these countries on the X-axis and the number of cases on the Y-axis gives us a Pearson’s r-value of 0.344 (p-value 0.504), which again changes from a negative correlation to a positive correlation after adjustment. The negative correlation between critical care beds and the fatality rate is justified, as intensive care unit (ICU) beds and ventilators are critical elements in the management of complicated cases. This importance of ventilators had previously been recognized in the study, which states that the provision of mechanical ventilators to developing countries has the unique potential to help make a dramatic improvement in the care of the world’s most vulnerable patients [19]. Mass testing in all suspected cases in Germany and South Korea, as laid down by WHO, could also be one of the reasons why they manage to reduce the number of new infections since it allows them to quickly identify possible outbreaks as early as possible for COVID-19, and we can also observe the pandemic curve for death rate to have bent quite effectively from the early days of the pandemic [20-21].

This approach proved to be a successful strategy to achieve a low fatality rate in both countries, as they used to test-admit/isolate-treat protocol. The National Health Service (NHS) of the UK used the Contain-Delay-Mitigate-Research strategy at the beginning and that turned out to be futile, with a possible association with a high case fatality rate [22].

**Figure 1:**
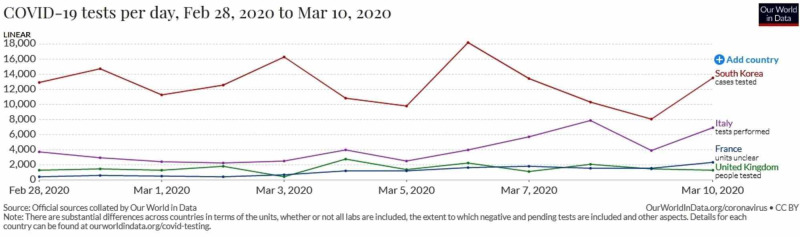
COVID-19 tests per day done in selected countries between February 28 and March 10, 2020 Adapted from: Our World in Data [23]

The number of tests done per day differed significantly among various countries from the beginning, as shown in Figure 1, and the day-by-day basis of COVID-19 tests per 1,000 people in Table 3, which clearly shows aggressive testing done to pick up cases early in South Korea while Italy, France, and the UK lagged way behind and, now, these three countries have a huge case fatality burden.

**Table 3:** COVID-19 tests done per 1,000 people in few selected countries between February 28 and March 10, 2020 Adapted from: Our World in Data [23]

Country Names	COVID-19 tests done per 1,000 people				
	Feb 28^th^	March 1^st^	March 5^th^	March 8^th^	March 10^th^
South Korea*	1.38	1.88	2.85	3.66	4.08
Italy	0.27	0.36	0.55	0.84	1.03
United Kingdom	0.13	0.17	0.27	0.35	0.39
France (data unclear though)	0.02	0.04	0.09	0.17	0.23

The COVID-19 epidemic has placed a significant burden on the health care system. This crisis has dramatically affected the delivery of critical care due to a lack of resources. This pandemic has exposed the skeleton of healthcare systems around the world, as well as the lack of preparedness of most of the countries to tackle a major crisis like this. The present search for estimating the contributing factors to COVID-19-related outcomes may not be likely to have been exhaustive. However, these findings have important implications for public health actions, as much of the world will witness a massive community epidemic of COVID-19 over the coming weeks and months. We try to make out a plan of action based on a few study reports that tried to address the gaps in NHS and the healthcare system as such in Table 3 [24-27].

**Table 4:** Possible measures to influence the course of the COVID-19 epidemic for the improved patient outcome ICU: intensive care unit

Problem	Possible measures or knowledge gaps to be addressed
Coordination	COVID-19 National Emergency Action Group should be established
Preparedness	Availability of adequate personal protective equipment (PPE) at the earliest; ventilators and ICU beds should be increased; Isolation wards with proper vigilance should be made with immediate effect
Strategy to be applied at the very onset	Test – admit/Isolate (explain symptoms when to get admitted or call for help) – treat
Isolation and admission	New framework by which prioritization criteria for scarce resources allocation need to be enforced such as admission criteria for high-risk patients or having moderate to severe symptoms; Positive cases should be advised for home isolation with proper care for 14 days and subsequent possible worsening of the symptoms and plan of action informed in the local language (to avoid the unwanted over-crowding in less available isolation wards)
Natural history of infection	Better understanding regarding the period of infectiousness and transmissibility and role of asymptomatic infectiousness and the degree to which this contributes to spread; accurate estimate the reproductive number in various outbreak settings
Rapid diagnosis and future research	Development of point-of-care diagnostic tests, validation of existing serological tests, and establishment of biobanks and serum panels of well-characterized COVID-19 sera to support such research efforts.
Treatment and research	Well-conducted studies are needed to assess, the magnitude (i.e. spectrum and severity) of various such life-threatening outbreak of infectious agents, the response to various therapeutic interventions, To address knowledge gaps about infection prevention and control in health-care settings; Support standardized, best evidence-based approach for clinical management and better outcomes and implement randomized, controlled trials for therapeutics and vaccines as promising agents emerge. Provision of new research facilities where health-care professionals can carry out the research for an infectious outbreak in the future.
Palliative care services	Scale up palliative care services to alleviate serious health-related suffering.
Post-peak period	The public will understandably wish to return to some semblance of normal life. Deep economic damage will be a powerful motivation to lift restrictions on personal freedoms. But to do so too early will lead inevitably to a second peak. The government must make the public aware of this phase.

## Conclusions

To conclude, the present analysis acts as just the beginning of the development of a thorough understanding of the impact of various epidemiological factors in coronavirus disease-infected patient outcomes. This will help the resource-limited regions to strategize a coordinated response for effectively managing and preparing for the emergence of this yet-to-be-known disease entity. Given the fact that this COVID-19 pandemic, for now, will have a long-term implication for all members of society, a collaborative effort among society, government, public health experts, and healthcare professionals will be needed to ensure efficient recovery from this pandemic disaster as early as possible.

## References

[1] Bedford J, Enria D, Giesecke J (2020). COVID- 19: towards controlling of a pandemic. Lancet.

[2] (2020). WHO. Coronavirus disease 2019 (‎COVID-19)‎: situation report, 78. https://apps.who.int/iris/handle/10665/331719.

[3] Tu YF, Chien CS, Yarmishyn AA (2020). A review of SARS-CoV-2 and the ongoing clinical trials. Int J Mol Sci.

[4] (2020). COVID- 19: too little, too late?. Lancet (London, England.

[5] (2020). The Economic Times. 2020. Global economy could shrink by almost 1% in 2020 due to COVID-19 pandemic: United Nations. https://economictimes.indiatimes.com/news/international/business/global-economy-could-shrink-by-almost-1-in-2020-due-to-covid-19-pandemic-united-nations/articleshow/74943235.cms.

[6] (2020). World Health Organization. Coronavirus disease 2019 (COVID-19). Situation report - 71. https://www.who.int/docs/default-source/coronaviruse/situation-reports/20200331-sitrep-71-covid-19.pdf.

[7] Onder G, Rezza G, Brusaferro S (2020). Case-fatality rate and characteristics of patients dying in relation to COVID-19 in Italy. JAMA.

[8] Wu Z, McGoogan JM (2020). Characteristics of and important lessons from the Coronavirus Disease 2019 (COVID-19) outbreak in China: summary of a report of 72314 cases from the Chinese Center for Disease Control and Prevention. JAMA.

[9] Nikolopoulos G, Bagos P, Lytras T, Bonovas S (2011). An ecological study of the determinants of differences in 2009 pandemic influenza mortality rates between countries in Europe. PLoS One.

[10] The OECD (2020). Health spending. https://data.oecd.org/healthres/health-spending.htm.

[11] Ghosal S, Sinha B, Sengupta S, Majumder M (2020). Frequency of testing for COVID 19 infection and the presence of higher number of available beds per country predict outcomes with the infection, not the GDP of the country - a descriptive statistical analysis [PREPRINT]. medRxiv.

[12] (2020). United States Census Bureau. Census.gov.

[13] (2020). Worldometer. Countries in the world by population (2020). https://www.worldometers.info/world-population/population-by-country/.

[14] McCarthy McCarthy, N. N., 2020 2020 (2020). The countries with the most critical care beds per capita. https://www.statista.com/chart/21105/number-of-critical-care-beds-per-100000-inhabitants/.

[15] (2020). Databank. World development indicators. Databank.worldbank.org.

[16] (2020). Worldometer. Coronavirus update (live). https://www.worldometers.info/coronavirus/#countries.

[17] (2020). Worldometers. Coronavirus testing criteria and numbers by country. https://www.worldometers.info/coronavirus/covid-19-testing/.

[18] Socscistatistics.com. (2019 (2020). Pearson correlation coefficient calculator. https://www.socscistatistics.com/tests/pearson/Default2.aspx.

[19] Krishnamoorthy V, Vavilala MS, Mock CN (2014). The need for ventilators in the developing world: an opportunity to improve care and save lives. J Glob Health.

[20] Who.int. 2020 (2020). WHO Director-General's opening remarks at the media briefing on COVID-19 - 16 March. https://www.who.int/dg/speeches/detail/who-director-general-s-opening-remarks-at-the-media-briefing-on-covid-19---16-march-2020.

[21] Our World in Data. 2020 (2020). Our World in Data. Daily confirmed COVID-19 deaths: are we bending the curve?. https://ourworldindata.org/grapher/covid-confirmed-daily-deaths-epidemiological-trajectory?time=2020-01-26..2020-04-11&country=CHN+DEU+KOR.

[22] Our World in Data. 2020 (2020). Our World in Data. Total COVID-19 tests per 1,000 people. https://ourworldindata.org/grapher/full-list-cumulative-total-tests-per-thousand?time=2020-02-28..2020-03-10&country=KOR+ITA+GBR+FRA.

[23] Horton R (2020). Offline: COVID-19 and the NHS—“a national scandal”. Lancet.

[24] Vergano M, Bertolini G, Giannini A (2020). Clinical ethics recommendations for the allocation of intensive care treatments, in exceptional, resource-limited circumstances:the Italian perspective during the COVID-19 epidemic. Crit Care.

[25] Mounk Y (2020). The extraordinary decisions facing Italian doctors. Atlantic. March 11.

[26] Kuhn A (2020). How a South Korean city is changing tactics to tamp down its COVID-19 surge. NPR. March 10.

[27] (2020). WHO. Critical preparedness, readiness and response actions for COVID-19. March 7.

